# A Mitochondria-Penetrating Peptide Exerts Potent Anti-Plasmodium Activity and Localizes at Parasites’ Mitochondria

**DOI:** 10.3390/antibiotics10121560

**Published:** 2021-12-20

**Authors:** Sangdao Somsri, Mathirut Mungthin, Natthaporn Klubthawee, Poom Adisakwattana, Warunee Hanpithakpong, Ratchaneewan Aunpad

**Affiliations:** 1 Graduate Program in Biomedical Sciences, Faculty of Allied Health Sciences, Thammasat University, Pathum Thani 12120, Thailand; ss_sangdao@hotmail.com (S.S.); p.natthaporn.k@gmail.com (N.K.); 2 Department of Parasitology, Phramongkutklao College of Medicine, Bangkok 10400, Thailand; mathirut@pmk.ac.th; 3 Department of Helminthology, Faculty of Tropical Medicine, Mahidol University, Bangkok 10400, Thailand; poom.adi@mahidol.edu; 4 Mahidol Oxford Tropical Medicine Research Unit, Faculty of Tropical Medicine, Mahidol University, Bangkok 10400, Thailand; Warunee@tropmedres.ac

**Keywords:** mitochondria-penetrating peptide, *Plasmodium falciparum*, antimalarial, localization

## Abstract

Mitochondria are considered a novel drug target as they play a key role in energy production and programmed cell death of eukaryotic cells. The mitochondria of malaria parasites differ from those of their vertebrate hosts, contributing to the drug selectivity and the development of antimalarial drugs. (F_x_r)_3_, a mitochondria-penetrating peptide or MPP, entered malaria-infected red cells without disrupting the membrane and subsequently killed the blood stage of *P. falciparum* parasites. The effects were more potent on the late stages than on the younger stages. Confocal microscopy showed that the (F_x_r)_3_ intensely localized at the parasite mitochondria. (F_x_r)_3_ highly affected both the lab-strain, chloroquine-resistant K1, and freshly isolated malaria parasites. (F_x_r)_3_ (1 ng/mL to 10 μg/mL) was rarely toxic towards various mammalian cells, i.e., mouse fibroblasts (L929), human leukocytes and erythrocytes. At a thousand times higher concentration (100 μg/mL) than that of the antimalarial activity, cytotoxicity and hemolytic activity of (F_x_r)_3_ were observed. Compared with the known antimalarial drug, atovaquone, (F_x_r)_3_ exhibited more rapid killing activity. This is the first report on antimalarial activity of (F_x_r)_3_, showing localization at parasites’ mitochondria.

## 1. Introduction

Malaria remains a life-threatening disease, causing millions of cases worldwide with around 405,000 deaths annually [1]. There are five different species of the *Plasmodium* parasite causing human malaria, namely *Plasmodium falciparum*, *Plasmodium vivax*, *Plasmodium ovale*, *Plasmodium malariae* and *Plasmodium knowlesi*. The most severe form is caused by *P. falciparum,* as it often leads to death and can be fatal not long after the first symptoms [2]. The increase and spread of multidrug-resistant *P. falciparum* have become major challenges to malaria treatment and are correlated with increases in morbidity and mortality in many malaria-endemic countries [1]. In response to this harsh predicament, artemisinin-based combination therapies (ACTs) have been recommended and widely established in malaria-endemic regions. Unfortunately, the recent Global Malaria Programme report pointed to artemisinin and ACT resistance causing significant delays in parasite clearance [3]. The development of novel antimalarial drugs and more effective therapy is urgently needed.

The *Plasmodium* mitochondrion contains the smallest eukaryotic mtDNA (6-kb), encoding only three genes for proteins and highly fragmented rRNA genes [4]. The differences in the mitochondria of malaria parasites from those of their vertebrate hosts allow for drug selectivity and potential targets for antimalarial drug development. Atovaquone is well known for malaria treatment, acts on the malaria mitochondrion, and has a very low frequency of side effects [5]. Even though the parasite has developed resistance to atovaquone, it maintains high efficiency when used in combination with proguanil for prophylaxis against malaria in endemic areas [6].

In the past ten years, it has been shown that several peptides can act on malarial parasites. The small linear angiotensin I and II-derived peptides exhibit antiplasmodial activity against the blood stage of both avian *P. gallinaceum* and *P. falciparum* [7,8]. Symplostatin 4 (Sym4), a marine-derived peptide, kills *P. falciparum* at nanomolar concentrations, generating a swollen food vacuole and inhibiting the initial stage of hemoglobin degradation [9]. Moreover, several host defense or antimicrobial peptides have been reported to disturb malaria homeostasis by disrupting parasite cellular membranes or interfering with key processes of parasite metabolism, and surprisingly kill the parasite [10,11]. One example is dermaseptin and its truncated derivatives, which exert anti-*P. falciparum* activity in the micromolar range by permeabilization of the cell membrane [12,13]. These antiplasmodial peptides, both natural and synthetic molecules, vary in size, charge, amino acid composition, hydrophobicity and secondary structure, and some of them appear to act selectively on infected erythrocytes and/or intraerythrocytic parasite membranes [11]. This implies that membrane-active peptides, such as cell-penetrating peptides (CPPs) or mitochondria-penetrating peptides (MPPs), which can translocate into cells without membrane damage, may be novel compounds for development as antimalarial agents.

Mitochondria-penetrating peptides or MPPs are a class of peptides with the ability to enter human cells and specifically target the mitochondria. The combination of two major characteristics, being cationic and lipophilic, drives the permeation of MPPs through the hydrophobic mitochondrial membrane [14]. The mechanism of MPPs depends on their concentration. The peptide binds to the membrane’s outer leaflet at low concentrations while crossing the hydrophobic bilayer and diffuses into both leaflets at high concentrations. Moreover, MPPs can cross to the inner leaflet without disturbing the lamellarity and transmembrane, which offer adequate energy for peptides to cross the hydrophobic core [15]. The characteristics and actions of MPPs lead to antimicrobial or antimalarial activities. One such short peptide, (L-cyclohexyl alanin-D-arginine)_3_ or (F_x_r)_3_, exhibited efficient and promising cell penetration and mitochondrial localization [14]. The objective of this study was to determine the antimalarial activity of (F_x_r)_3_ and elucidate its localization in *P. falciparum*. Our findings suggest that (F_x_r)_3_ might be developed as a new antimalarial agent for use alone or in combination with current antimalarial agents.

## 2. Results

### 2.1. (F_x_r)_3_ Showed No Toxicity toward L929 Mouse Fibroblast Cells

**Figure 1:**
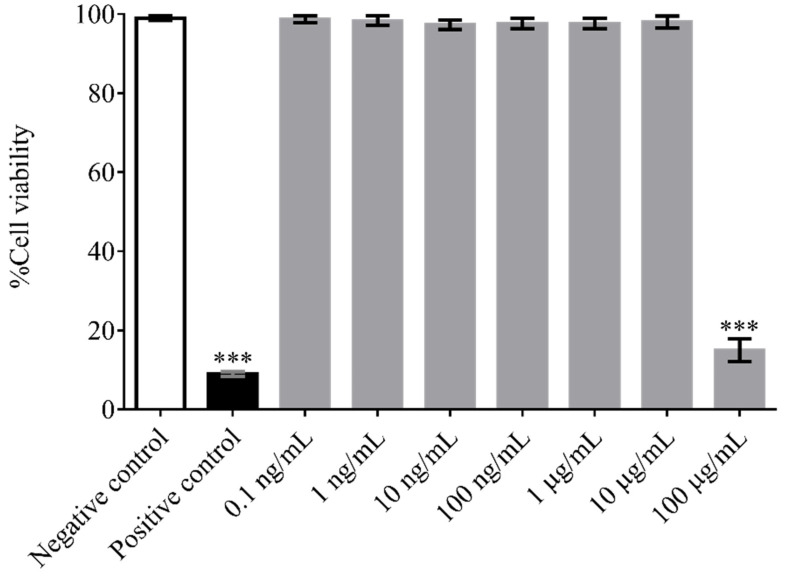
The viability of L929 mouse fibroblast cells after treatment with different concentrations of (F_x_r)_3_. Data were obtained from three individual experiments. Stars indicate a significant difference compared with negative control (*p* < 0.05). Untreated cells and cells treated with 20% DMSO were used as negative and positive controls, respectively.

The cytotoxicity activity of (F_x_r)_3_ toward L929 mouse fibroblast cells was determined using the MTT (3-(4,5-dimethylthiazol-2-yl)-2,5-diphenyltetrazolium bromide, a tetrazole) assay. As shown in Figure 1, at concentrations from 1 ng/mL to 10 µg/mL, (F_x_r)_3_ showed no toxicity with cell viabilities of more than 99%, which is comparable to that of the negative control (untreated cells). By contrast, at the highest concentration of (F_x_r)_3_ tested (100 µg/mL), the viability of these fibroblast cells was less than 10%.

### 2.2. (F_x_r)_3_ Demonstrated Very Low Toxicity against Human Peripheral White and Red Blood Cells

**Figure 2:**
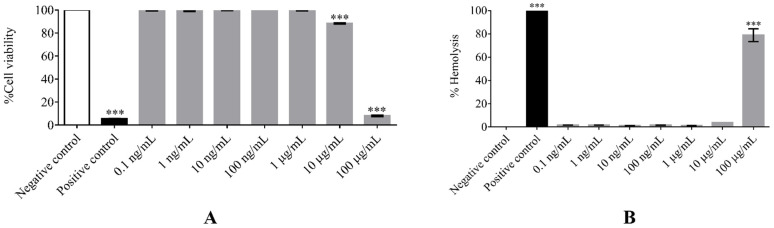
The cell viability of human PBMCs (**A**) and red blood cells (**B**) after treatment with (F_x_r)_3_ at different concentrations. Data were obtained from three individual experiments. Stars indicate a significant difference compared with the negative control (*p* < 0.05).

The toxicity of (F_x_r)_3_ against human peripheral white blood (PBMCs) and red blood cells was determined by MTT and hemolysis assays, respectively. The results showed that the viability of human PBMCs was not significantly affected by (F_x_r)_3_ at concentrations from 1 ng/mL to 1 µg/mL when compared to that of negative controls (Figure 2A). This was in accordance with the results of hemolytic activity determinations. However, at a concentration of 10 µg/mL, there was less toxicity toward red blood cells, and this was not significantly different from that of negative controls (Figure 2B). At the highest concentration tested (100 µg/mL), (F_x_r)_3_ exhibited a lethal effect on both human PBMCs and red blood cells.

### 2.3. (F_x_r)_3_ Exhibited Antimalarial Activity in Nano Range

**Figure 3:**
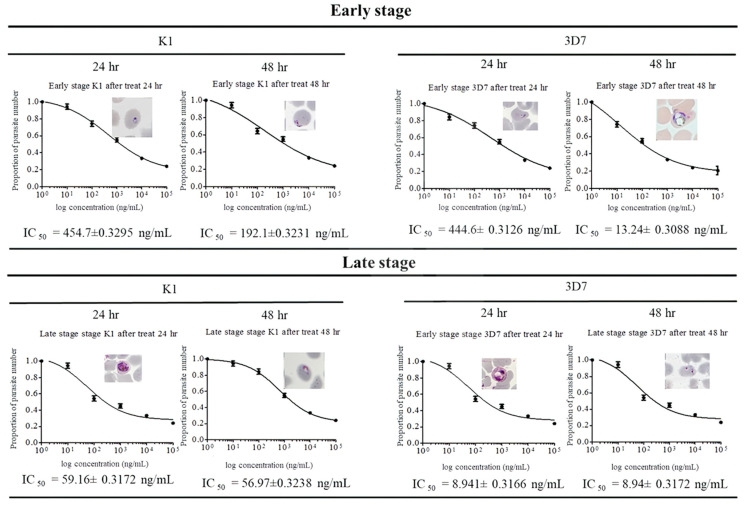
In vitro drug sensitivity assay of (F_x_r)_3_ against early- and late-stage malarial parasite strains K1 and 3D7. All parasites were stained by Giemsa and observed under a light microscope (100× oil-immersion).

Antimalarial activity of (F_x_r)_3_ was determined against chloroquine-sensitive (3D7) and resistant (K1) strains of *Plasmodium falciparum,* as well as two field isolates (J1 and MR4). Due to the different forms of parasite mitochondria, the effect of (F_x_r)_3_ was evaluated in both early- and late-stage parasites (24 and 48 h). IC_50_ was measured, and parasite morphology was observed under light microscopy. After incubation of (F_x_r)_3_ with early-stage parasites for 24 h, IC_50_ of strain K1 and 3D7 were 454.7 and 444.6 ng/mL, respectively (Figure 3). Both K1 and 3D7 showed similar morphology, with rounded chromatin and large vacuoles. After 48 h of incubation, parasites did not develop into the trophozoite and schizont stages and displayed a large vacuole with compact chromatin. The IC_50_ values of (F_x_r)_3_ against early-stage parasites after 48-h incubation were 192.1 ng/mL and 13.24 ng/mL for strains K1 and 3D7, respectively. The IC_50_ of (F_x_r)_3_ against early-stage of both strains, K1 and 3D7, at 24-h incubation was significantly different from that after 48-h incubation (*p* < 0.001).

After a 24-h treatment of (F_x_r)_3_ toward late-stage parasites, the IC_50_’s were 59.16 and 8.94 ng/mL for strains K1 and 3D7, respectively. Microscopy revealed that the parasites did not develop into the mature schizont stage and showed a large vacuole with compact chromatin. After continuing incubation to 48 h, the IC_50_ of (F_x_r)_3_ toward strain 3D7 was not significantly different from that after the 24-h incubation (*p* = 0.997). On the contrary, the IC_50_ of 24-h incubation of strain K1 was significantly different from that after 48-h incubation (*p* = 0.001). The morphology of parasites after a 48-h incubation (under light microscopy) was of atypical rings with compact chromatin. By statistical analysis of both strains, the IC_50_ of (F_x_r)_3_ showed significant differences between early- and late-stages, and also 24- and 48-h incubations (*p* < 0.001).

The antimalarial activity of (F_x_r)_3_ against two field isolates, J1 and MR4, was determined with both early- and late-stage parasites (24 and 48 h), as shown in Table 1. After 48 h of incubation of both early- and late-stage parasites with (F_x_r)_3_, the IC_50_ of both field isolated strains were significantly lower than those at 24 h. By statistical analysis, the IC_50_ of (F_x_r)_3_ showed significant differences between early- and late-stage, and also 24- and 48-h incubations (*p* < 0.001).

### 2.4. (F_x_r)_3_ Localized at Parasite’s Mitochondria

**Figure 4:**
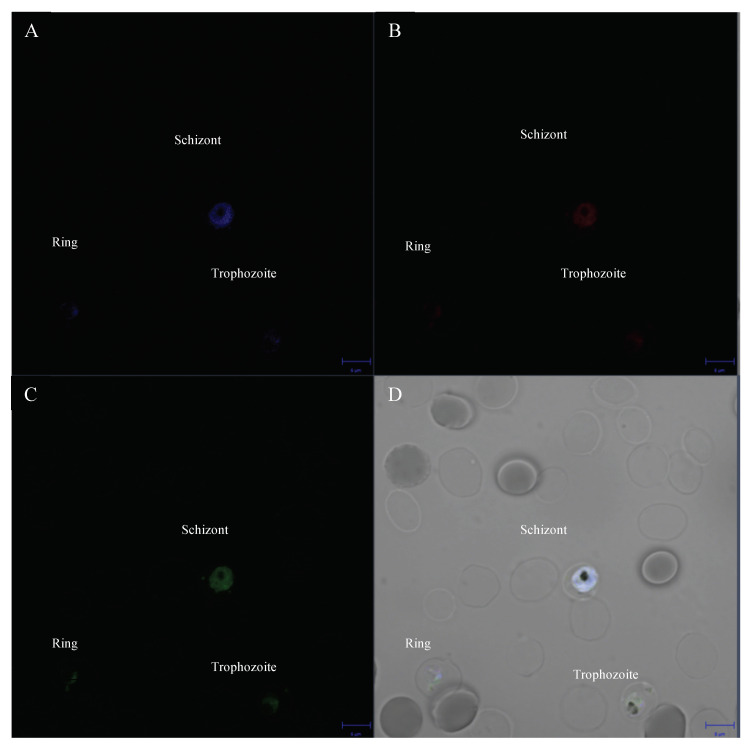
(F_x_r)_3_ localization as observed by confocal microscopy. Bright field image shows all stages of the parasite living in red blood cells (**D**). Hoechst 33,342 (**A**), TM-(F_x_r)_3_ (**B**) and mitotracker green (**C**) staining, representing DNA, TM-(F_x_r)_3_ and mitochondria, respectively.

The localization of (F_x_r)_3_ was observed in strain K1 under confocal microscopy using (F_x_r)_3_ linked with TAMRA dye or TM-(F_x_r)_3_. The parasite’s nucleus and mitochondria were stained with fluorescence dye Hoechst 33,342 and mitotracker green, respectively. Hoechst 33,342 was used to stain the parasite nucleus demonstrating a live malarial parasite, while active mitochondria were stained with mitotracker green. As shown in Figure 4A, the schizont stage of strain K1 displayed a deep blue color in the middle of the cell, while light staining was observed in ring and trophozoite stages representing living parasite cells. By observing with mitotracker green, TM-(F_x_r)_3_ showed localization of (F_x_r)_3_ at the mitochondria of the cell. Late-stage parasites (trophozoite and schizont) displayed a higher intensity of TM-(F_x_r)_3_ than did the ring stage.

### 2.5. (F_x_r)_3_ Killed Malarial Parasites More Rapidly than ATQ

**Figure 5:**
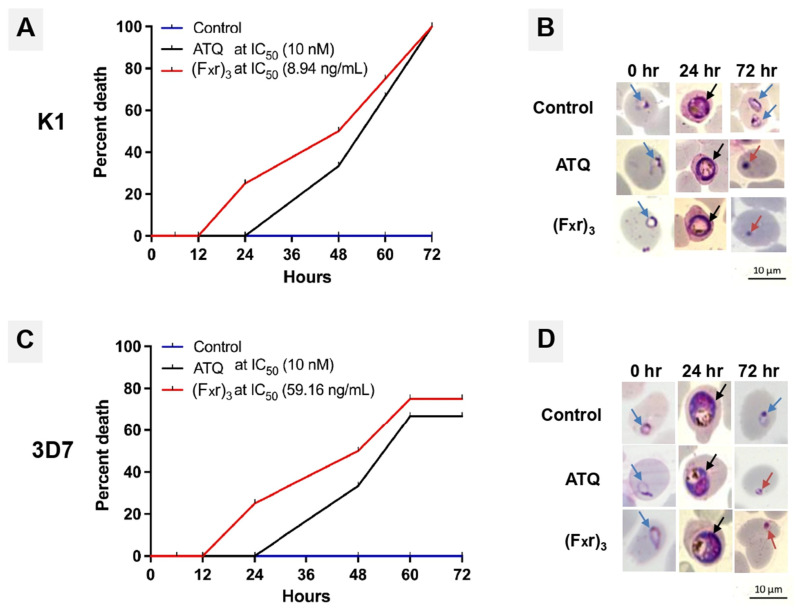
The percent death of strain K1 (**A**) and 3D7 (**C**) and Giemsa stain of strain K1 (**B**) and 3D7 (**D**) during incubation with (F_x_r)_3_ and ATQ for 72 h. Blue, black, and red arrows indicate ring, trophozoite and dead parasite, respectively.

The killing rate of malarial parasite strain K1 and 3D7 after incubation with (F_x_r)_3_ was compared to that with atovaquone (ATQ) by determination of malarial cell death at 72 h (Figure 5). At 12 h, there was no death of ring-stage parasites (0% death) in both the ATQ-treated and (F_x_r)_3_-treated groups. Parasite death was observed at 24 h of incubation with (F_x_r)_3_ (25% death), while ATQ-treated group showed no parasite death after 24 h. The parasite death at 24 and 48 h of the (F_x_r)_3_-treated groups were significantly different from that of ATQ-treated groups (*p* < 0.05). At 48 h, the parasite developed to the late trophozoite and schizont stages. Parasite re-infection led to new rings after 60 h, and the new rings were growing to a mature stage at 72 h. The death of parasites treated with (F_x_r)_3_ was not significantly different from that of ATQ-treated parasites after 60 and 72 h of incubation (*p* > 0.05).

## 3. Discussion

Malaria mitochondrion could be an attractive and selective target for the development of antimalarial drugs, as it plays a key role in energy production and is different from those of their vertebrate hosts. Even though the outer membrane of a mitochondrion is semi-permeable, allowing free access of small and large molecules up to 5 kDa, the inner membrane maintains a very tight barrier to the transport of many small and large molecules (including drugs) to the mitochondrial matrix [16]. Mitochondria-penetrating peptides or MPPs have been developed to accelerate the transport of molecules into the human mitochondrion [14]. MPPs can target and penetrate the mitochondria of active eukaryotic cells by two major properties, being cationic and lipophilic. These characteristics may explain the antiplasmodial activity of our peptide. In order to exploit MPPs as antimalarial agents, they must also be able to pass through the red blood cell membrane without disruption or destruction. After passing through this membrane, they must penetrate the parasite membrane and specifically target the mitochondria. However, mitochondria of malaria exhibit some extraordinary evolutionary and functional features, such as the membrane composition, which is different from that of eukaryotic cells [5]. Moreover, the mitochondrial characteristics of each stage of the parasite are different, so the ability of MPPs to act on parasite mitochondrion must be observed. In this study, a promising mitochondria-targeting peptide, known as (L-cyclohexyl alanine-D-arginine)_3_ or (F_x_r)_3_, was selected for elucidating the antimalarial activity of MPPs.

(F_x_r)_3_ exhibited antimalarial activity against both chloroquine-sensitive (3D7) and resistant (K1) strains of *P. falciparum* with, notably, more effective antiplasmodial activity toward late-stage parasites as shown by IC_50_ values. This might be due to more active mitochondria in early-stage parasites. There was a report on the stage-specific presence of cristae inside *P. falciparum* mitochondrion. In ring-stage parasites, the mitochondrion is elongated, acristate with low electron density. In trophozoites, the mitochondrion is significantly larger with an unchanged appearance from that of the ring stage. In mature schizonts, each daughter merozoite harbors one small, acristate, electron-lucent mitochondrion in close proximity to one four-membrane-bound apicoplast [17]. There are discrepancies in the IC_50_ results between strains K1 and 3D7. The antimalarial drug susceptibility of both strains revealed that they showed different sensitivity to antimalarial drugs, including chloroquine, piperine, mefloquine and artesunate. Strain K1 is highly resistant to chloroquine (IC_50_ is 115.0 nM), while strain 3D7 is very sensitive (IC_50_ of 3.9 nM). In contrast, strain K1 exhibited lower sensitivity toward other antimalarial drugs such as piperine and artesunate. The IC_50_ of strains K1 and 3D7 against mefloquine were 4.22 nM and 14.6 nM, respectively. The IC_50_ of strains K1 and 3D7 against artesunate were 1.06 nM and 2.48 nM, respectively [18]. It has been noted that the lowest IC_50_ of (F_x_r)_3_ toward chloroquine-resistant *P. falciparum*, K1 strain, was only 59.16 ng/mL. The IC_50_ values are within the nM range and are comparable to the IC_50_ values of established drugs toward different laboratory and field-isolated strains of *P. falciparum* such as chloroquine (9.8–744 nM) and artemisinin monomer (6.6–42.5 nM) and their derivatives: dihydroartemisinin or DHA (2.09–14.8 nM) and artesunic acid or ARS (0.82 nM) [19]. In general, it can be said that (F_x_r)_3_ exhibits high antimalarial activities against chloroquine-resistant strains of *P. falciparum*.

The action of (F_x_r)_3_ was confirmed by cellular localization at the mitochondria of living malaria parasites. (F_x_r)_3_ exhibited efficient cellular uptake and specific mitochondrial localization in human cells such as HeLa and MRC-5 cells [14]. Late-stage parasites (trophozoite and schizont) displayed higher amounts of (F_x_r)_3_ localized in mitochondria than did ring stage (early-stage), and this might be due to the large mitochondrion of trophozoite and schizont compared to that of the ring stage [17]. This was in accordance with the higher antimalarial activity of (F_x_r)_3_ toward parasites observed in late-stage. The antimalarial action of (F_x_r)_3_ might be similar to that of atovaquone (ATQ), which caused a collapse of mitochondrial membrane potential. ATQ targets cytochrome *b*, which plays a role in electron transport during mitochondrial respiration [20] and functions by inhibiting mitochondrial electron transport and depolarizing malarial mitochondria leading to cell damage and death, while showing no effect on mammalian mitochondrial membrane potential [5]. However, ATQ is expensive and subject to relatively facile development of resistance [21]. The target of (F_x_r)_3_ in the parasite’s mitochondrion is still unelucidated and needs to be identified in order to pave the way for the development of new antimalarial agents. MPPs have been widely applied for gene and drug delivery to mitochondria, especially in conjunction with anticancer drugs such as cisplatin [known as MPP-cisplatin conjugate (mtPt)], which can induce apoptosis by damaging mitochondrial DNA without damaging nuclear DNA and overcomes cisplatin resistance in cancer cells [22].

(F_x_r)_3_ could be an alternative choice for development as an antimalarial drug attacking mitochondria or used in combination with ATQ. Antagonism between ATQ and antimalarial drugs (quinolines and artesunate) has been previously reported [23]. Additionally, atovaquone-proguanil or malarone is prescribed as a fixed-dose prophylactic agent for travelers in malaria-endemic areas and used for treating uncomplicated malaria [21]. The combination study of ATQ and (F_x_r)_3_ will be further elucidated to identify a potential benefit of antimitochondrial effects. By comparing the death rate of parasites after different time intervals, (F_x_r)_3_ was found to kill *P. falciparum* strain K1 more rapidly than did ATQ with significant differences at 24 and 48 h. The combination of ATQ and (F_x_r)_3_, focused on the same target, could rapidly kill parasites throughout their eukaryotic life cycle. There are reports of substantial genetic differentiation among mitochondrial genomes of *P. vivax* collected from different regions [24] and the difficulty in vitro culturing due to the low amounts and mixed stages of the parasite. Therefore, knowledge of the parasite’s biology was far less than that of *P. falciparum*. However, the usefulness of (F_x_r)_3_ as an anti-vivax agent still needs to be explored, as drugs with the same target (such as ATQ) can kill *P. vivax* with an IC_50_ value of 30 nM [25].

As evaluated by cytotoxicity assays, (F_x_r)_3_ showed very low toxicity toward normal mouse fibroblast L929 and human peripheral white blood cells at concentrations ranging from 1 ng/mL to 10 µg/mL. Moreover, it did not exhibit any toxicity toward red blood cells. This might be due to the cationic and lyophilic characteristics of (F_x_r)_3_ facilitating cellular uptake and permeation of the hydrophobic mitochondrial membrane without cytotoxic activity [14]. Even though (F_x_r)_3_ at the highest concentration tested (100 µg/mL) exhibited cytotoxicity and hemolytic activity, this concentration was around one thousand times higher than that of antimalarial activity. Several studies found that MPPs have low toxicity both in vitro and in vivo [14,26,27], suggesting the applicability of MPPs as delivery vectors and/or bioactive compounds. Besides toxicity, the sensitivity of peptides to protease degradation is a critical concern. Hemolysis is a hallmark of malaria infection and can release proteases. Moreover, there are several proteases released from parasites/lysing host cells and present in plasma/serum. Fortunately, the sequence of (F_x_r)_3_, which is composed of L-cyclohexylalanine and an artificial arginine residue, is not cleaved by endogenous protease [28].

Our results demonstrated that (F_x_r)_3_ can pass through the red blood cell membrane without disruption or destruction, and subsequently kill the blood stage of *P. falciparum*. (F_x_r)_3_ showed more potent antimalarial activity toward late-stage (trophozoite and schizont) parasites, consistent with high intensity of (F_x_r)_3_ localized in the parasites’ mitochondria observed by confocal microscopy. Moreover, (F_x_r)_3_ exhibited high antimalarial activity against the chloroquine-resistant K1 strain of *P. falciparum* and showed very low toxicity toward normal mouse fibroblast L929 cells, human peripheral white blood cells (PBMCs) and red blood cells. To our knowledge, there is no study on the sensitivity and specificity of (F_x_r)_3_ toward the malaria parasite, *P. falciparum*. This is the first report on the antimalarial activity of (F_x_r)_3_. (F_x_r)_3_ or other MPPs might be developed as new antimalarial agents or used in combination with current antimalarial agents targeting the mitochondrion, such as atovaquone.

## 4. Materials and Methods

### 4.1. Culture of Parasite

*Plasmodium falciparum* strains 3D7 (CQ-sensitive clone), K1 (CQ-resistance clone) and two field-isolated strains (J1 and MR4) were used in this study. They were kindly provided by the Department of Parasitology, Phramongkutklao College of Medicine, Thailand. All isolate strains were continuously cultured using standard methods with modifications [29]. For parasite culture, human blood group O^+^ RBCs were used, and white blood cells were removed by washing with RPMI 1640. Complete culture medium was prepared by adding 10 mL of 0.5% albumax II (Invitrogen, Waltham, MA, USA) to each 200 mL aliquot of stock RPMI 1640 medium (Gibco URL, Waltham, MA, USA) containing 2.05 mM L-glutamine, 25 mM HEPES, 21 mM sodium bicarbonate and gentamycin (5 g/mL). A 5% parasitemia and 5% hematocrit were maintained under 5% CO_2_, 3% O_2_ and 92% N2 at 37 °C. Five percent D-sorbitol (Sigma, St. Louis, MO, USA) was used for parasite synchronization by adding 10 mL of 5% D-sorbitol per 1 mL packed red cells and incubating at 37 °C for 30 min, and after that washing with RPMI1640 at 2000× *g* for 5 min.

### 4.2. Mitochondria-Penetrating Peptide

A short peptide (L-cyclohexyl alanine-D-arginine)_3_ or (F_x_r)_3_, composed of alternating hydrophobic cyclohexylalanine (F_x_) and D-arginine (r), was used as a mitochondria-penetrating peptide (MPP) in this study [14]. It was kindly provided by Professor Dr. Shana O. Kelley, University of Toronto, Canada. Synthesis of the peptide was performed by using solid-phase synthesis on Rink amide resin (0.6–0.7mmol/g, 100,200 mesh) (NovaBiochem, Darmstadt, Germany). The peptide was purified by reversed-phase high-pressure liquid chromatography, and the purity was greater than or equal to 95%. Liquid chromatography-mass spectrometry (LC-MS) coupled with electron-spray ionization was used to confirm the identity of the conjugate.

### 4.3. Cytotoxicity Determination

The cytotoxic activity of (F_x_r)_3_ was assessed with the L929 mouse skin fibroblast cell line. Cells were cultured in RPMI 1640 medium with 10% fetal bovine serum, 2 mM of L-glutamine, 100 U/mL of penicillin and 100 mg/mL of streptomycin at 37 °C. Cells were incubated at 37 °C in a humidified incubator with 5% CO_2_. Confluent cells were treated with 0.5% trypsin solution for detachment, diluted with 10% serum-containing medium. The cells were then plated in 96-well culture plates at 30,000 cells/cm^2^. Culture plates were incubated for 24 h at 37 °C in a humidified incubator with 5% CO2 prior to stimulation of the cells with the compound. After that, L929 was incubated with ten-fold serial dilutions of (F_x_r)_3_ with concentrations ranging from 0.1 ng/mL-100 μg/mL. Viability of the cells was determined by 3-(4,5-dimethylthiazol-2-yl)-2,5-diphenyl tetrazolium or MTT assay. After removing the medium, cells were incubated with MTT solution (5 mg/mL in PBS) for 4 h and the resulting formazan was solubilized with DMSO (100 μL). Formazan absorbance was measured at 550 nm using an automated microplate reader. Cell viability was expressed as a percentage of the control culture value. Untreated cells and cells treated with 20% DMSO were used as negative and positive controls, respectively. All experiments were carried out in triplicate.

### 4.4. Peripheral White Blood Cell Toxicity Assay

Blood group O+ human PBMCs were isolated from fresh heparinized blood by Lymphoprep (r = 1.077 g/mL; Axis-Shield, Oslo, Norway). Lymphocytes were isolated from buffy coats by Lymphoprep followed by staining with Trypan Blue and number counting to determine cell survival (live and dead cells) with 96% live cell. Human lymphocytes were conserved in RPMI 1640 medium until use, no longer than two weeks. Cells were plated on 96-well plates, 1000 cells per well, and co-incubated with ten-fold serial dilution of (F_x_r)_3_ with concentrations ranging from 0.1 ng/mL–100 μg/mL, at 37 °C for 24 h. Supernatants were collected after incubation, and the viability of the cells was determined by MTT assay as described above.

### 4.5. Hemolytic Assay

The human red blood cells (hRBCs) (2% vol/vol) were incubated with ten-fold serial dilutions of (F_x_r)_3_ with concentrations ranging from 0.1 ng/mL–100 μg/mL at 37 °C for 24 h. Supernatants were collected after centrifugation at 2000× *g* for 10 min, and the absorbance was determined by spectrophotometry at 540 nm (Labomed Inc, Los Angeles, CA, USA). The values were converted to % hemolysis by comparing to the control (100% hydrolysis) using distilled water (DW).

### 4.6. In Vitro Drug Sensitivity Assay

Sensitivities of *P. falciparum* to (F_x_r)_3_ and atovaquone (ATQ) were investigated based on the incorporation of [^3^H] hypoxanthine into parasite nucleic acids or radioisotopic technique [30]. The level of radioactivity uptake was used as the index of parasite growth. All strains were maintained in continuous culture at 5% parasite, 5% hematocrit using type O^+^ human erythrocytes in RPMI 1640 with 0.5% Albumax (Invitrogen), 25 mM HEPES (Sigma), under a 3% O_2_, 4% CO_2_ and 93% N2 gas mixture. Parasites (1% hematocrit) were incubated with ten-fold serial dilutions of (F_x_r)_3_ or ATQ in the microtiter plate at 37 °C. At the end of the incubation period, the plates were removed from the chamber, and a final concentration of 50 µCi [^3^H] hypoxanthine (NEN, Boston, MA, USA) was added to each well. The plates were gently agitated to ensure adequate mixing of the parasite/drug solutions with the radiolabeled hypoxanthine and thereafter placed back into the incubation chamber and gassed for 5 min prior to incubation at 37 °C for 24 h. After incubation, the assay plates were harvested onto filtermats (Wallac A printed Filltermats, Turku, Finland), using a Tomtec March III M semi-automatic harvester. The filtermats were subsequently removed from the harvester and dried at 60 °C in an oven prior to scintillation counting. Each dry filtermat was placed inside a plastic sample bag, and 1 mL of a Beta-plate Scint (Wallac, Turku, Finland) was added on top of it. The bag was then sealed before being heated in a 1495-021 Microsealer (Wallac, Finland). Each filtermat was placed in a cassette, and the radioactivity measured using a 1450 Micro-Beta Trilux liquid scintillation and luminescence counter (Wallac, Turku, Finland).

### 4.7. (F_x_r)_3_ Localization

The late stage of *P. falciparum* strain K1 was incubated with (F_x_r)_3_ linked with TAMRA or TM-(F_x_r)_3_ at concentration of the IC50 for 4 h. Hoechst 3342 (Sigma, Ronkonkoma, NY, USA), at a final concentration of 10 μg/mL, was added into the cultures in order to determine the live parasite cells. Mitotracker green (Cell Signalling, Danvers, MA, USA) was added to achieve a final concentration of 400 nM and incubated for 30 min at 37 °C. Then, the cells were washed two times with PBS pH 7.4. Images were taken with confocal laser scanning (ZEISS LSM 700). The excitation wavelength for visualization for Hoechst 33,342 was 355 nm, and emissions were collected at 461 nM. The excitation wavelength for visualization of the mitotracker green was 490 nm, and emissions were collected at 516 nM. The excitation wavelength for visualization TAMRA or (F_x_r)_3_ was 555 nm, and emissions were collected at 580 nM.

### 4.8. Data Analysis

All experiments were performed in triplicate, and the results are presented as mean ± standard deviation (SD). The data were analyzed by one-way analysis of variance (ANOVA) with GraphPad Prism version 5; *p*-value < 0.05 was considered statistically significant.

## Figures and Tables

**Table 1:** In vitro drug sensitivity assay of (F_x_r)_3_ against early- and late-stage field isolated malarial parasites.

	Strain	IC_50_ (ng/mL)	
		24 h Incubation	48 h Incubation
Early-Stage	J1	426.2 ± 0.42	116.0 ± 0.44
	MR4	804.1 ± 0.42	51.86 ± 0.42
Late-stage	J1	24.94 ± 0.42	9.33 ± 0.38
	MR4	113.2 ± 0.44	33.26 ± 0.41

## Data Availability

Data is contained within the article. The underlying raw data are available from the corresponding author on request.
